# Hypothalamic-pituitary hormones will be affected by the interaction between 5q13-14-rs2239670 (CARTPT) gene variants and diet in different obesity phenotypes

**DOI:** 10.1186/s13104-021-05857-5

**Published:** 2021-12-07

**Authors:** Mahsa Mahmoudinezhad, Mahdieh Abbasalizad-Farhangi, Houman Kahroba

**Affiliations:** 1 Department of Community Nutrition, Faculty of Nutrition and Food Science, Tabriz University of Medical Sciences, Tabriz, Iran; 2 Drug Applied Research Center, Tabriz University of Medical Sciences, Attar-neishabouri Ave, Golgasht St, 5165665931 Tabriz, Iran; 3 Centre for Environmental Sciences, Hasselt University, Hasselt, Belgium; 4 Department of Toxicogenomics, GROW School of Oncology and Development Biology, Maastricht University, Maastricht, The Netherlands

**Keywords:** CARTPT, Diet quality indices, Cardio-metabolic risk factors, Obesity

## Abstract

**Objective:**

Evidence show that cocaine and amphetamine regulated transcript-prepropeptide (CART-PT) gene variants may affect obesity related traits, but little is known about its end points. In the current study, we aimed to evaluate the interaction of CARTPT gene polymorphism with diet quality indices including dietary approaches to stop hypertension (DASH) and Mediterranean diet score (MDS) on cardio-metabolic risk factors. This cross sectional study recruited 288 apparently healthy obese individuals. Diet quality indices including DASH and MDS were evaluated using semi quantitative food frequency questionnaire (FFQ). Polymerase chain reaction-restriction fragment length polymorphism (PCR–RFLP) was used for CARTPT genotypes.

**Results:**

No significant differences was reported for general characteristics and biochemical parameters across genotypes except for QUICKI among females (P = 0.01) and it was higher in heterozygous genotype. There was significant CARTPT-DASH interactions affecting serum fasting glucose level (P = 0.049). However, in relation to CERTPT-MDS interactions, the highest level of insulin (P = 0.003) and HOMA-IR (P = 0.003) values were shown among AA carriers in high adherence to MDS, while AA carriers in high compliance to MDS experienced decreased level of QUICKI (P = 0.001).

**Supplementary Information:**

The online version contains supplementary material available at 10.1186/s13104-021-05857-5.

## Introduction

The prevalence of obesity has increased to pandemic proportion over the past years [1–3]. Also Iran has experienced increasing trend of obesity in the recent years and it has become a threat for Iranian population [4]. This increasing trend requires new and effective strategies to halt its prevalence as a priorities of world health organization (WHO) [3]. To this end, numerous studies have been conducted just with a one-dimensional perspective about the effect of genetic signatures or nutrient intake on the risk of obesity alone, which have not been more effective. So taking to account of the gene variants effects combined with dietary intakes about cardio-metabolic risk factors may provide a new insight with most promising strategies for personalized nutrition [5, 6]. Although obesity related risk factors are modifiable with healthy dietary patterns but modification of this phenomena may be affected by genetic predisposition which can affect individual’s response to dietary interventions [1, 7]. Genome wide association studies (GWAS) identified susceptible loci that contributes to cardio-metabolic risk factors across genotypes, and in this sense the role of cocaine and amphetamine regulated transcript prepropeptide (CART-PT) gene became a topic of recent interest [7, 8]. CART-PT, co-expressed with pro-opiomelanocortin (POMC) in the anorexigenic neurons of hypothalamus arcuate nucleus, encoding CART protein [9–12]. In addition, it is noteworthy that CART-PT is involved in energy balance, feeding behaviors and obesity [9–11, 13, 14]. On the other hand, evidence indicate that anorexigenic and orexigenic neuropeptides which are expressing proopiomelanocortin (POMC) and agouti related peptide (AgRP) respectively, have profound effects in energy balance too. Moreover, alpha-melanocyte stimulating hormone (α- MSH), one of the derivative of POMC along with CART, inhibit energy intake and AgRP leads to increased appetite and suppressed metabolic rate [9, 10, 15]. Likewise, several dietary indices have been developed to estimate the healthfulness of dietary patterns. Of these, dietary approaches to stop hypertension (DASH) score and Mediterranean diet score (MDS) emerge a better clarification of synergistic and interactive effects of nutrients. According to epidemiological evidence, high adherence to DASH and MDS, offer a beneficial effect about metabolic risk factors [16]. Previous studies indicated that more adherence to these dietary indices is associated with decreased risk of chronic diseases and obesity related traits [17–19]. Accordingly, this study aimed to better capture the interaction between genetic variants of CART-PT and nutrient intake in relation to metabolic factors and provide a complementary approach regarding gene-diet interactions.

## Main text

### Methods

The present cross-sectional study conducted in Tabriz, Iran. This study recruited apparently healthy obese people. Accordingly, 288 eligible patients who met the following inclusion criteria enrolled to study: adults aged 20–50 years old, BMI > 30 kg/m^2^. In contrast, patients with history of chronic diseases such as kidney disorders, cardiovascular diseases, type 2 diabetes mellitus, pregnancy, lactation and alcohol abuse were excluded and finally 147 men and 141 women completed the study. Patients who were eager to participate in the study were asked to respond the questions related to demographic characteristics as well as socio-economic status (SES). The study protocol was approved by the ethics committee of Tabriz University of Medical Sciences (Ethics number: IR.TBZMED.REC.1397.266).

Anthropometric measurements performed by a highly skilled person including: height, weight and waist circumference (WC). Weight assessments was done in accordance to standards with a light clothes by an electronic balance (Seca, Germany) nearest to 100 g. Height measurements was conducted without shoes using a stadiometer with the accuracy of 0.1 cm. Waist circumference (WC) was measured at the point between the last rib and the iliac crest using a non-stretchable tape to the nearest 0.5 cm [20]. Systolic and diastolic blood pressure were assessed in a relaxing position and body composition was measured by body composition analyzer BC-418-Tanita (United Kingdom).

Blood sample (10 cc) was obtained from all patients after overnight fasting. Then they were centrifuged at 4 °C, 3000 rpm for 10 min and stored at − 80 °C. Total cholesterol (TC), fasting serum glucose, high density lipoprotein-cholesterol (HDL-C) and triglyceride (TG) measured by commercial kit (Pars Azmoon, commercial kit, Tehran, Iran). Also we calculated low-density lipoprotein cholesterol (LDL-C) using Friedewald equation [21]. Serum insulin level was measured by similar ELIZA kit. Homeostasis model assessment insulin resistance index (HOMA-IR) and quantitative insulin sensitivity check index (QUICKI) values were computed too [22]. In addition, plasma α-MSH and AgRP were detected using commercially enzyme-linked immunosorbent assay (ELISA) kits (Bioassay Technology Laboratory, Shanghai Korean Biotech, Shanghai City, China) based on manufacture’s protocol. The minimum level of detection for α-MSH and AgRP levels were 5.07 ng/L and 1.03 pg/ml, respectively.

Habitual dietary intake of individuals over the preceding years was evaluated using a validated 132-item semi-quantitative food frequency questionnaire (FFQ) [23, 24]. Participants were asked to report their usual consumption frequency and amounts of foods based on daily, weekly, monthly and yearly. Finally, the reported frequency for each food item was computed as a daily intake. Portion sizes of the daily intakes were converted to grams per day using household measurements and nutrient intake calculated using Iranian food composition table (FCT) [25]. In DASH dietary pattern, consumption of some components are encouraged to be increased and some suggested to be minimized. It is constructed of 8 components emphasizing on high intakes of vegetables, fruits, legumes, nuts, whole grains and low-fat dairy products while diminished intakes of red and processed meats, sweetened beverages and sodium. According to intake ranking, each component classified into quintiles and zero points was awarded to emphasized components to high intake and a score of 5 for low recommended ones. Finally, the DASH diet score can be measured from total score obtained [26]. Also, we aimed to evaluate MDS as an indicator of adherence to Mediterranean diet which, was constructed by Trichopoulou et al. in the present study [27]. It focuses on 9 components including: legumes, vegetables, cereals, fruits and nuts, meats, fish, dairy products, alcohol and the ratio of monounsaturated fatty acid (MUFA) to saturated fatty acid (SFA). MDS considers 0 or 1 score for each component with respect to sex-specific median as the cutoff. In that case, for person whose consumption of beneficial components (vegetables, legumes, fruits, cereal, nuts and fish) was below the median awarded 0 and equal or above the median assigned 1. In contrast, for components with deleterious properties such as: meat, poultry, and dairy products, which are rarely nonfat or low-fat in Greece, a value of 1 was assigned for consumption below the median and 0 for those consumption was at or above the median [27]. In accordance to sum of all components score, MDS score will range from 0 (low adherence) to 9 (high adherence) finally [27].

**Fig. 1:**
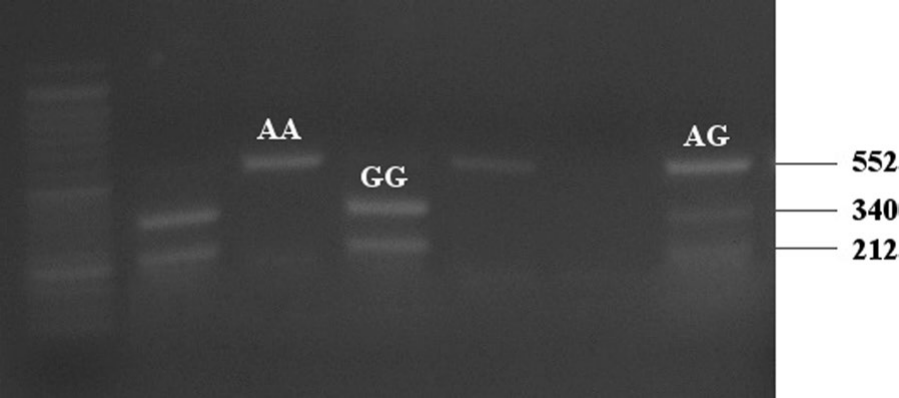
Genotyping of CART rs2239670 variant by Apa-I PCR–RFLP analysis. M: 50 bp DNA ladder

DNA extraction procedures conducted to all blood samples. The CARTPT rs2239670 SNP was genotyped by polymerase chain reaction-restriction fragment length polymorphism (PCR–RFLP) technique. PCR was performed as follow: denaturation for 30 s at 94 °C, annealing for 30 s at 60 °C, and extension for 20 s at 72 °C, for 35 cycles. The forward and backward primers were 5′-CCTGCTGCTGATGCTACCTCT-3′ and 5′-GCGCTTCGATCTGCAACACAC-3′, respectively. Three genotypes were detected: homozygous AA (552 bp), heterozygous AG (212, 340 and 552 bp) and homozygous GG (340 and 212 bp) (Fig. 1 and Additional file 1: Fig. S1).

Statistical analyses were performed using SPSS software, Version 22. We used Mean ± SD representing normally distributed continuous variables and the median (25th and 75th percentile) for not normally distributed continuous variables. One-way analysis of variance (ANOVA) and chi-square tests were used for reporting general characteristics of participants across CARTPT genotypes. The interaction between dietary indices and CART gene variants identified by ANCOVA multivariate model with adjustment to confounders. P-values < 0.05 was considered statistically significant.

### Results

**Fig. 2:**
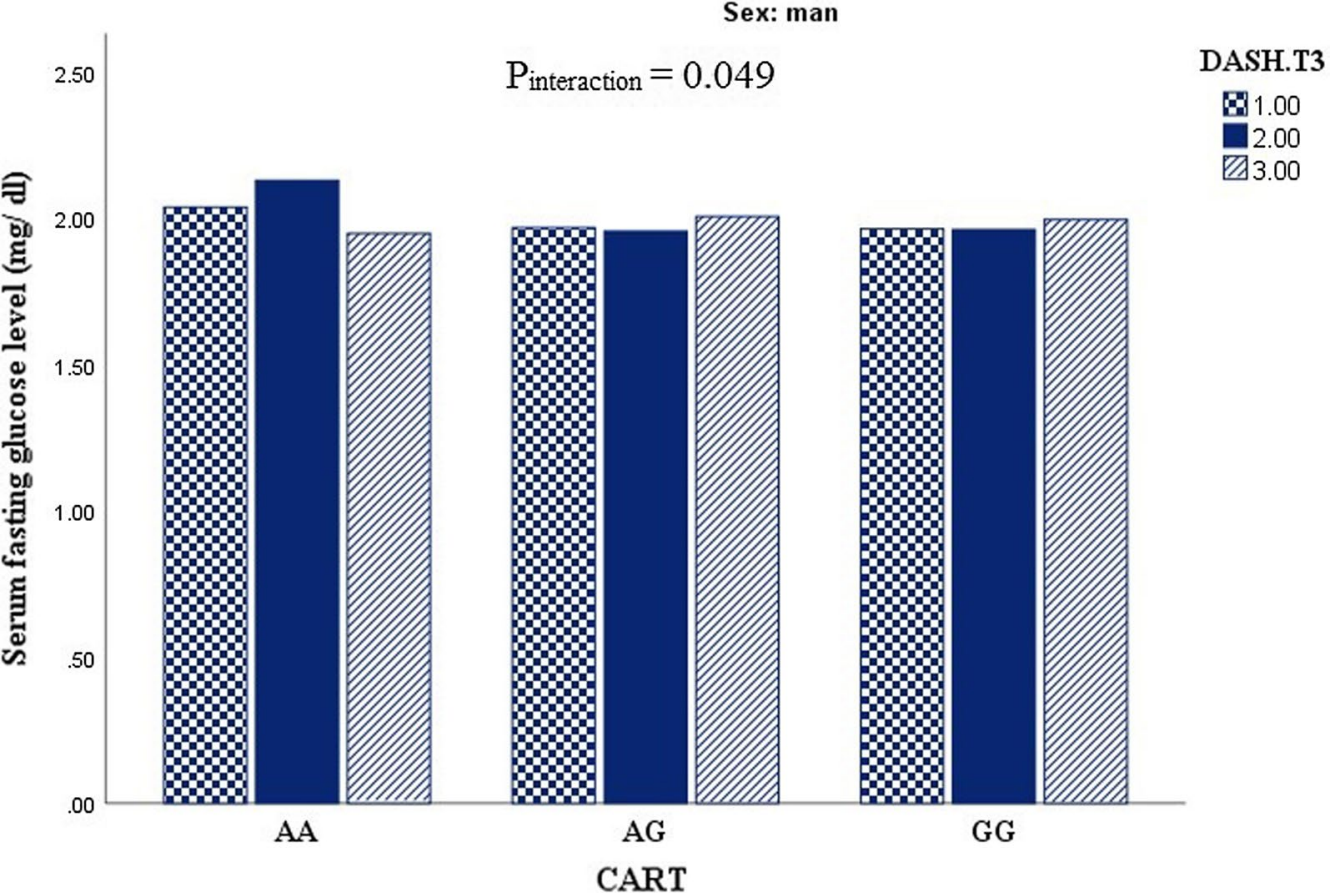
Interaction between CARTPT rs2239670 and DASH score on serum fasting glucose level among men (P _interaction_ = 0.049)

**Fig. 3:**
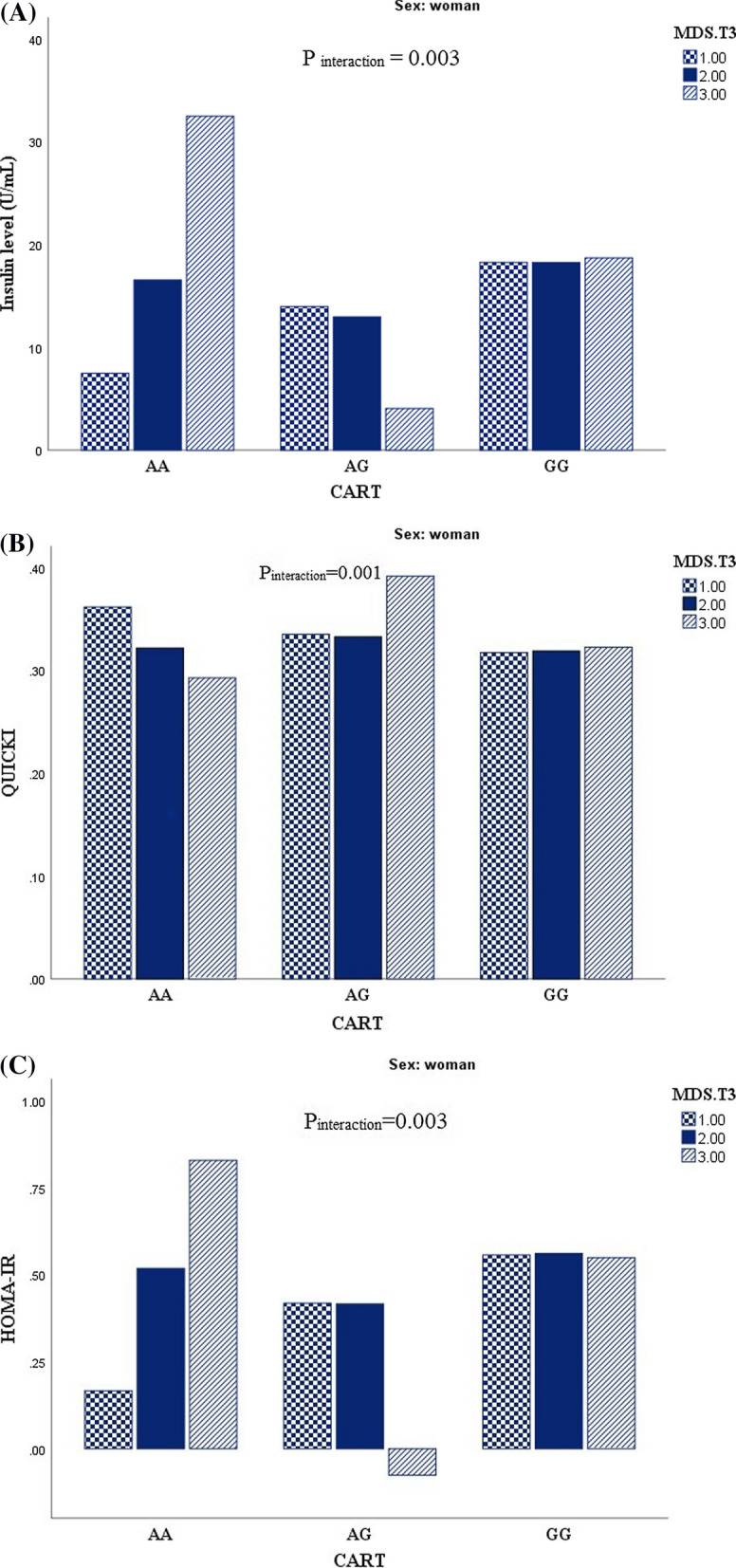
Interaction between CARTPT rs2239670 and MDS on insulin level (P _interaction_ = 0.003) (**A**), QUICKI (P _interaction_ = 0.003) (**B**) and HOMA-IR (P _interaction_ = 0.003) (**C**) among women

This cross sectional study recruited 288 apparently healthy obese individuals including 51% men and 49% women. General characteristics and biochemical parameters of study participants across CARTPT rs2239670 genotypes are presented in Table 1. In comparison of biochemical parameters, QUICKI value showed a significant difference according to genotypes in female group (P = 0.01), which shows a high level of QUICKI in heterozygous genotype in comparison to other genotypes. This association remained significant even after adjustment for potential confounders. Due to gene-diet interaction analysis, serum fasting glucose levels may be affected by CARTPT-DASH interaction (P_interaction_ = 0.049) among men (Fig. 2). Accordingly, high adherence to DASH score in men contribute to decreasing serum fasting glucose level in third tertile of DASH score in comparison to those in second tertile. Additionally, men with AA genotypes have the highest level of serum fasting glucose level in the second tertile of DASH score adherence. Also, CARTPT-MDS interaction effects can be shown on insulin level (P_interaction_ = 0.003), QUICKI (P_interaction_ = 0.001) and HOMA-IR (P_interaction_ = 0.003) among women (Fig. 3). Despite of high adherence to MDS in AA homozygous genotype an increasing level of insulin and HOMA-IR was shown among female group; likewise the highest insulin and HOMA-IR levels appeared in the last tertile of MDS score. While AG genotype somehow could attenuate this effect and the lowest level of insulin and HOMA-IR was shown in high adherence to MDS. In contrast, females with homozygous genotype for A allele had the lowest mean of QUICKI in high adherence to MDS, and in relation to QUICKI value, females who were assigned to third tertile of MDS showed the highest level of QUICKI.

**Table 1:** General characteristics and biochemical characteristics of study participants across the CARTPT rs2239670 genotypes

	Women				Men			
	AA	AG	GG	P*	AA	AG	GG	P*
Age (y)	35.85 (7.89)	37.44 (9.06)	38.41 (7.99)	0.56	40.40 (5.36)	42.27 ( 7.24)	37.24 (6.42)	**0.01**
BMI (kg/m^2)^	34.75 (4.22)	34.89 (3.63)	36.03 (4.46)	0.45	33.14 (2.38)	35.09 (5.52)	33.63 (2.53)	0.23
FM	38.24 (7.56)	36.07 (6.00)	39.53 (9.10)	0.30	24.80 (1.60)	32.18 (10.44)	28.71 (6.41)	0.11
WC (cm)	103.00 (10.71)	100.34 (8.91)	105.85 (10.08)	0.11	111.60 (3.13)	115.30 (11.95)	112.63 (6.33)	0.39
WHR	0.85 (0.05)	0.86 (0.04)	0.88 (0.06)	0.08	1.00 (0.03)	0.98 (0.04)	0.99 (0.03)	0.58
BMR (kcal)	1605.29 (209.01)	1523.15 (134.36)	1605.77 (142.95)	0.08	2151.81 (146.68)	2152.03 (225.11)	2222.62 (360.05)	0.77
HC (cm)	120.57 (9.80)	116.61 (8.31)	119.56 (9.27)	0.40	111.20 (2.16)	117.00 (8.63)	113.76 (6.24)	0.11
SBP (mmHg)	113.21 (18.57)	114.27 (15.64)	114.06 (14.82)	0.97	107.00 (8.36)	114.27 (28.43)	117.53 (12.87)	0.35
DBP (mmHg)	75.07 (13.11)	79.16 (10.03)	76.18 (11.84)	0.55	67.00 (9.08)	74.11 (19.46)	76.30 (10.08)	0.25
LDL-C (mg/dl)	122.82 (28.47)	124.32 (37.89)	116.66 (34.00)	0.64	125.68 (27.63)	114.48 (24.87)	122.07 (29.43)	0.56
HDL (mg/dl)	47.50 (10.25)	50.05 (7.87)	46.40 (9.37)	0.34	43.00 (6.74)	39.22 (6.87)	43.00 (6.89)	0.12
Cholesterol (mg/dl)	190.50 (33.59)	191.83 (36.94)	185.71 (36.83)	0.78	199.80 (32.87)	180.05 (27.49)	191.16 (32.40)	0.31
TG (mg/dl)	100.85 (34.30)	87.27 (34.73)	113.26 (43.26)	0.05	155.60 (53.83)	131.72 (62.35)	130.49 (70.65)	0.73
Glucose (mg/dl)	93.00 (80–121)	86.5 (70–124)	89.00 (71–122)	0.36	99.00 (81–183)	90.00 (78–193)	93.95 (77–141)	0.15
Insulin, U/ml	12.15 (4–33)	10.90 (3.10–27.30)	16.40 (3.20–4.20)	0.23	28.9 (8.9–29.9)	12.25 (4.10–30.90)	11.50 (3.30–31.10)	0.86
HOMA-IR	2.76 (0.82–8.64)	2.58 (0.60–6.13)	3.93 (0.63–8.80)	0.33	6.63 (1.78–8.05)	2.76 (0.86–8.05)	2.69 (0.64–7.37)	0.37
QUICKI	0.32 (0.03)	0.34 (0.03)	0.31 (0.02)	**0.01**	0.30 (0.02)	0.32 (0.02)	0.32 (0.02)	0.14
AgRP (pg/ml)	20.85 (17.10 – 90.00)	23.75 (16.50–75.00)	22.15 (12.60–65.00)	0.35	23.9 (18.40–89.00)	27.00 (17.80–63.00)	26.15 (14.70–94.00)	0.74
α-MSH (ng/L)	141.25 (105.6–680.0)	147.75 (119.1–659.0)	138.5 (98.60–624.0)	0.67	139.00 (128.5–689.0)	166.65 (116.1–620.00)	156.40 (95.10–684.00)	0.74

Data are presented as mean (SD) or median (min, max)

BMI: body mass index; WC: waist circumference; SBP: systolic blood pressure; DBP: diastolic blood pressure; LDL-C: low density lipoprotein cholesterol; HDL: high-density lipoprotein; TG: triglyceride; HOMA-IR: homeostasis model assessment of insulin resistance; QUICKI: quantitative insulin sensitivity check index; AgRP: agouti-related protein; α-MSH: alpha melanocyte stimulating hormone

*Analysis of variance for continuous variables and χ^2^ test for categorical variables

### Discussion

To the best of authors' knowledge, the current study discusses about the interactions between CARTPT rs2239670 and dietary indices including DASH score and MDS in relation to metabolic risk factors for the first time. The genotype and allele distribution in this study, is similar to the studies conducted in Korea [28] and Malaysia [13]. Indeed, different demographic features and dietary patterns may explain this discrepancies. In accordance to our results, there was no significant differences between genotypes in relation to SBP and DBP and anthropometric parameters (WC, WHR and BMI) in Yeo study’s [13]. Previous study conducted in Japanese population, showed an association between obesity and polymorphism of CARTPT gene in the promoter region [29], while another variant in the 3’-UTR, C1442G (rs1800926) was not associated with obesity in Pima Indians [30]. Moreover, Guérardel A. designed a study to evaluate the effect of CART gene polymorphisms and its association with obesity related traits in Caucasian population, which represented an association between SNP-3608T>C (rs7379701) and obesity [31]. Furthermore, no association was reported about lipid profile and CART SNP like our study [31].

As one of the most outstanding findings of the present study, we can mention the significant interactions of CARTPT rs2239670 with DASH score on fasting serum glucose level among males even after adjusting for potential confounders. DASH dietary pattern tended to improve hyperglycemia in type 1 diabetic patients [32], which are in agreement with our results that subjects assigned to third tertile of DASH score represented lower level of glucose in comparison to second tertile. Also we documented an interesting significant interaction between CARTPT rs2239670 and MDS in term of insulin, QUICKI and HOMA-IR levels in women. The present study attempted to clarify the effects of dietary factors as a modulator, which can modify the association of CARTPT gene polymorphism and cardio-metabolic risk factors, therefore, it can affects the genetic susceptibility to a variety of chronic diseases such as obesity. AA-carriers had the highest level of insulin and HOMA-IR, in spite of high adherence to MDS. In contrast, increased adherence to MDS could well demonstrate its beneficial effects in decreasing insulin and HOMA-IR levels in AG carriers, which indicates the modifying effects of G allele attenuating genetic predisposition to metabolic risk factors. It is the result of individual variability in changing the ease and extent of alteration in metabolic risk factors. Whereas, Abiemo reported that high consistency with MDS is associated with low insulin level in non-diabetic subjects [33]. In line with the present study, normo-glycemic and diabetic patients revealed lower levels of insulin and HOMA-IR in the upper tertile of MDS compared to lower tertiles of diet score in Panagiotakos et al.’s study [34].

In general, the present study revealed that having AA genotype makes subjects more prone to have increased levels of insulin and HOMA-IR even in high accordance to MDS. Indeed, this genotype makes subjects more vulnerable to cardio-metabolic risk factors.

### Limitations

Each study has its own limitations. First, cross sectional design doesn’t provide a platform to investigate cause and effect relationships. Second, it seems that underestimation in the report of FFQ may affect the results. Third, according to individual’s variability, it is better to be repeated in other population.

## Supplementary Information

**Additional file 1: Fig. S1.**Full length, original unprocessed gels of CART rs2239670 genotyping by Apa-I PCR-RFLP analysis. M: 50 bp DNA ladder.

## Data Availability

Participants in this study did not agree to the public sharing of their data so supporting data is not available.

## Abbreviations

BMI: Body mass index
GWAS: Genome wide association studies
SNP: Single nucleotide polymorphisms
SBP: Systolic blood pressure
DBP: Diastolic blood pressure
TG: Triglyceride
TC: Total cholesterol
HDL-C: High-density lipoprotein cholesterol
LDL-C: Low-density lipoprotein cholesterol
HOMA-IR: Homeostasis model assessment-insulin resistance index
QUICKI: Quantitative insulin sensitivity check index
Ag-RP: Agouti-related peptide
α-MSH: α-Melanocyte-stimulating hormone
FFQ: Food frequency questionnaire
FCT: Food composition table
PCR–RFLP: Polymerase chain reaction-restricted length polymorphism
