# Supplementary material for: Hypothalamic-pituitary hormones will be affected by the interaction between 5q13-14-rs2239670 (CARTPT) gene variants and diet in different obesity phenotypes
Source: BMC Res Notes. 2021 Dec 7;14:443. doi: 10.1186/s13104-021-05857-5 (PMC8649315; doi:10.1186/s13104-021-05857-5)
Supplement: Supplementary file 1 — Additional file 1: Fig. S1.Full length, original unprocessed gels of CART rs2239670 genotyping by Apa-I PCR-RFLP analysis. M: 50 bp DNA ladder. [file 13104_2021_5857_MOESM1_ESM.docx]

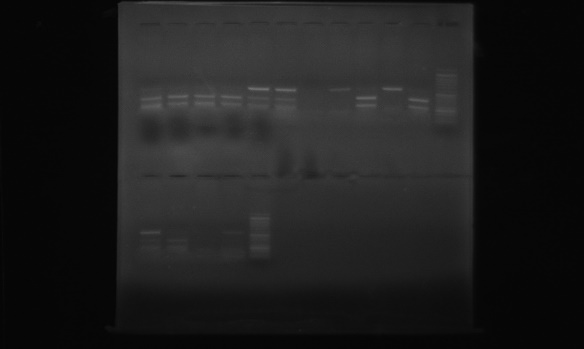

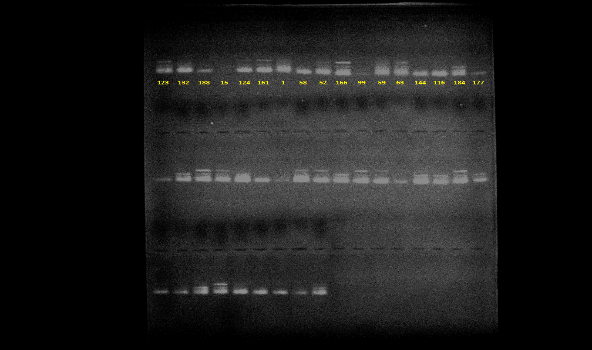

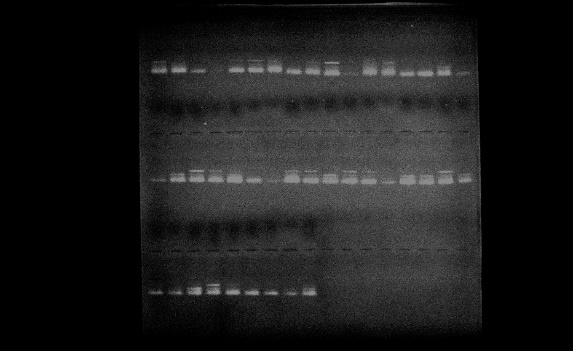


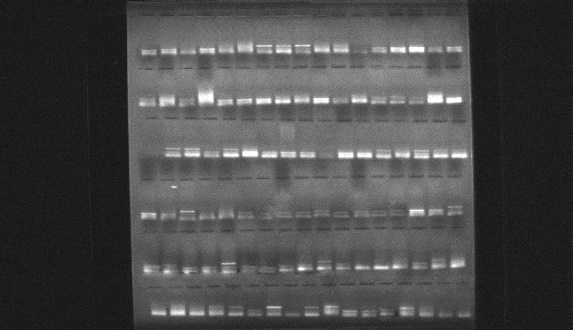

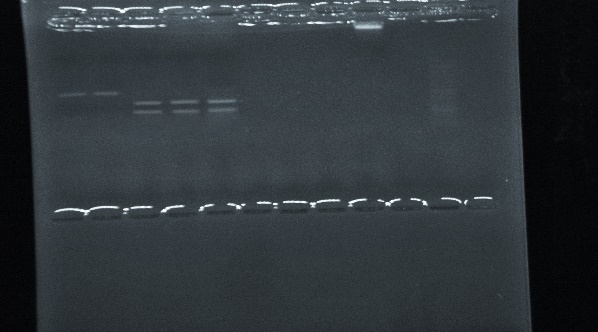

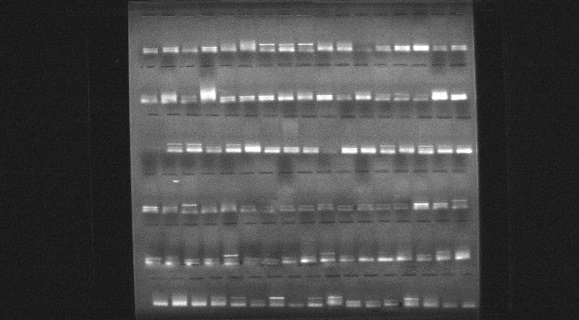


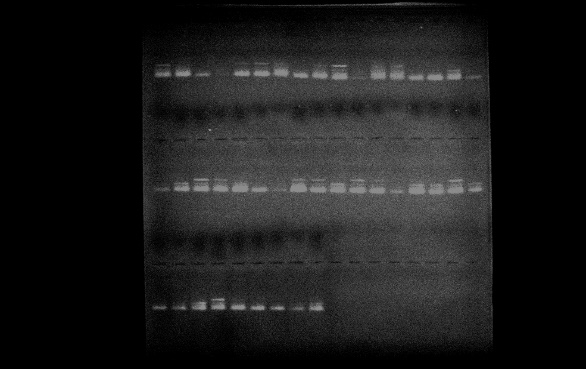

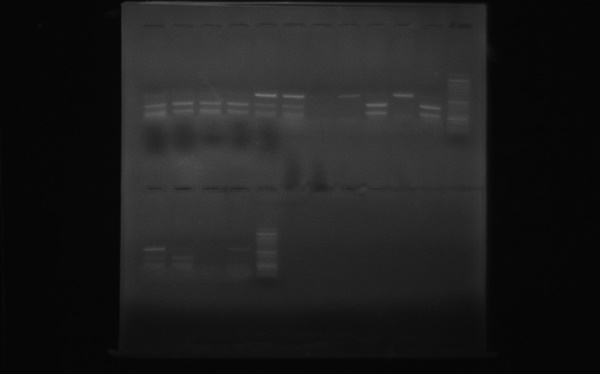

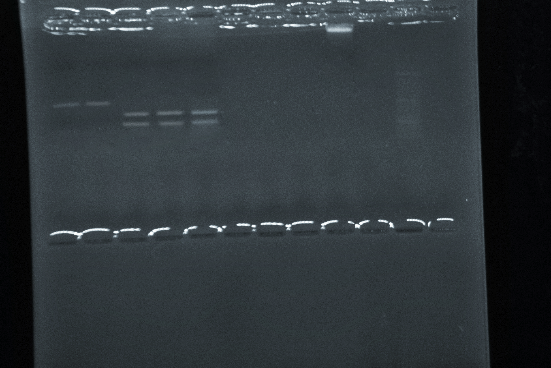


**Supplementary Figure 1.** Full length, original unprocessed gels of CART rs2239670 genotyping by Apa-I PCR-RFLP analysis. M: 50 bp DNA ladder.
